# Quantifying large carnivore predation relative to human harvest on moose in an intensively managed boreal ecosystem

**DOI:** 10.1002/eap.70000

**Published:** 2025-02-11

**Authors:** Håkan Sand, Barbara Zimmermann, Petter Wabakken, Ane Eriksen, Camilla Wikenros

**Affiliations:** ^1^ Grimsö Wildlife Research Station, Department of Ecology Swedish University of Agricultural Sciences Riddarhyttan Sweden; ^2^ Faculty of Applied Ecology, Agricultural Sciences and Biotechnology University of Inland Norway Koppang Norway

**Keywords:** anthropogenic impact, brown bear, harvest, kill rate, moose, predation rate, sustainable management, wolf

## Abstract

The return of large carnivores to areas with strong anthropogenic impact often results in conflicts among different interest groups. One cause of conflict is that large carnivores compete with humans for wild game species. In Scandinavia, the recolonization of wolves (*Canis lupus*) and brown bears (*Ursus arctos*) has important ramifications for the harvest of an ungulate species with high economic and recreational value, the moose. We estimated wolf and brown bear predation rates on moose (*Alces alces*) relative to harvest, natural causes of death, and vehicle collisions within 20 wolf territories. We used data on multi‐season kill rates of wolves and brown bears on moose combined with wolf territory sizes and estimates of the population density of brown bears and moose. Wolf predation rate on moose was not related to the density of moose, wolf pack size, nor kill rate but was positively related to wolf density and strongly negatively related to the abundance of moose within wolf territories. Estimated annual wolf and brown bear predation rates averaged 8.6% (range 2.8%–16.9%) and 2.3% (range 0%–12.7%) respectively, among wolf territories, whereas estimated annual harvest rates averaged 17.5% (range 8.1%–33.1%). In wolf territories with relatively high bear densities, the combined predation rates from wolves and brown bears exceeded harvest rates. Across wolf territories, harvest rates were not related to wolf predation rates or to the combined predation rates from wolves and brown bears, indicating that large carnivore predation and harvest were not compensatory to each other at this spatial level. The recolonization of these large carnivores in the Scandinavian boreal forest ecosystem may have small to significant consequences for the sustainable management of moose populations depending on the local conditions of both wolves, brown bears, and moose. Comparison of annual mortality rates for moose in our study in Scandinavia with corresponding data from areas with lower anthropogenic impact (Alaska) shows lower total mortality rates in Scandinavia. This likely results from a different age and sex composition of moose killed by wolves and brown bears versus harvest, in combination with a significant difference in the relative importance of these mortality factors between areas.

## INTRODUCTION

The impact of predation on prey populations depends on multiple factors such as the type and number of both predator and prey species, the magnitude of predation, the age and sex structure of killed animals, and the degree of compensatory mortality (Gervasi et al., 2012; Peterson et al., 2014). In addition, environmental factors can modulate the impact of predation on prey populations (Elmhagen et al., 2010; Melis et al., 2009; Vucetich & Peterson, 2004).

Central to quantifying the impact of predation on prey population dynamics is the estimation of predation rate on prey populations (Messier, 1994; Skogland, 1991; Taylor, 1984), which is defined as the proportion of the prey population killed during some defined time period (Taylor, 1984). For example, using empirical data from three study sites of wolves and prey, Vucetich et al. (2011) showed that predation rates were strongly positively related to the predator‐to‐prey ratio and that this ratio explained a significant portion of the variation in prey population growth rate for two of the three study sites in North America.

Most studies on the impact of large carnivores on ungulate prey have been performed in North America where moose (*Alces alces*), elk (*Cervus elaphus*), or white‐tailed deer (*Odocoileus virginianus*) constituted the main prey of large carnivores such as wolves and brown bears (Ballard et al., 2001; Boertje et al., 2009, 2010; Gasaway et al., 1983, 1992; Messier, 1994; NRC, 1997). Results from these and other studies suggest that wolf and brown bear populations that are not, or only weakly, regulated by harvest have the potential to limit populations of moose and elk to relatively low levels, that is, restrict growth of prey populations (Boertje et al., 2009, 2010; Hamlin et al., 2009; Jedrzejewski et al., 2002). However, a recent literature review followed by a meta‐analysis of both management and natural experiments of predator control showed relatively weak and mixed effects of predation on ungulate demography (Clark & Hebblewhite, 2021).

Common to most of these studies is that they have been performed in ecosystems with relatively low anthropogenic impact on both predators and prey, for example, harvest. In contrast to these studies, many areas in the world today harbor ungulate prey populations that to various extent are limited and shaped structurally by human harvest (Melis et al., 2009; Peterson et al., 2014; Ripple & Beschta, 2012). In some of these areas, large carnivores have returned relatively recently (Chapron et al., 2014), sometimes resulting in both predator and prey populations being limited by human harvest (Chapron et al., 2016; Liberg et al., 2020; Suutarinen & Kojola, 2017; Tuominen et al., 2023; Wikenros et al., 2015, 2020).

Large carnivore control is often motivated by the anticipation of reduced yields from ungulate harvest due to predation by large carnivores (Gervasi et al., 2012; Jonzén et al., 2013; Nilsen et al., 2005; NRC, 1997). Alternatively, large carnivores such as wolves may be less important for ungulate population growth as compared to human harvest, climatic variability, and habitat alteration effects (Clark & Hebblewhite, 2021; Harding et al., 2020). For example, in a study of the recovering Yellowstone wolf population, an observed decrease in the elk population was suggested to be better explained by human harvest and climatic effects than by predation by wolves (Vucetich et al., 2005). However, this view was later questioned by studies claiming that the combined predation by wolves and brown bears was the main source of mortality (Hamlin et al., 2009).

Because prey, predator, and environmental conditions show large variation among populations and areas, further insight into the numerical impact of large carnivores on prey populations is needed on a case‐by‐case level and where the impact of predation can be compared with that of harvest and environmental factors. Few studies have so far presented quantitative empirical estimates of the relative contribution of large carnivore predation and human harvest to total mortality or population growth of ungulates (but see Brodie et al., 2013; Garrott et al., 2009; Gasaway et al., 1983, 1992).

The Scandinavian ecosystem is strongly shaped by human resource utilization on several levels in the food web, and large carnivores such as wolves and brown bears have relatively recently recolonized significant parts of the peninsula (Kindberg et al., 2011; Ordiz et al., 2015; Wabakken et al., 2001). The most controversial of the two carnivore species, the wolf (Mech, 2017), is strongly regulated by both legal harvest and poaching (Liberg, Chapron, et al. 2012; Liberg et al., 2020), resulting in a slowly growing population characterized by a patchy distribution of territories and a high turnover (Mattisson et al., 2013; Tallian et al., 2021). Wolf predation in this system and its impact on their main prey (moose) will therefore mainly be confined to distinct areas of the moose population that spatially overlap with existing wolf territories (Rodríguez‐Recio et al., 2022; Sand, Vucetich, et al. 2012; Wikenros et al., 2020; Zimmermann, 2014). In contrast, the brown bear population has been recolonizing the peninsula for almost a century and shows a more continuous distribution with a few core population areas characterized by a relatively high density (Bischof et al., 2020).

The recolonization of wolves and brown bears in Scandinavia has been met with strong resistance from certain groups in human society, especially in rural areas where people live closer to these carnivores as compared to more urban areas (Ericsson & Heberlein, 2003; Pohja‐Mykrä, 2016; Skogen & Krange, 2003; von Essen et al., 2017). One of the major sources of conflict is the anticipated competition between large carnivores and humans for a valuable resource (Jonzén et al., 2013; Wikenros et al., 2015, 2020), the moose, of which the harvest has both great economic and recreational value (Boman et al., 2011; Storaas et al., 2001). In Scandinavia, harvest is the main regulating factor (>90% of mortality) of the moose population in areas without large carnivores (Ericsson & Wallin, 2001; Rönnegård et al., 2008; Solberg et al., 1999). However, because moose also cause severe damage to commercially valuable plants (mainly Scots pine (*Pinus sylvestris*); Bergqvist et al., 2014; Hörnberg, 2001; Månsson, 2009), moose harvest is considered an important management tool to limit and regulate moose population size in order not only to reduce browsing damages on commercially valuable forest (Gicquel et al., 2020; Lavsund et al., 2003; Liberg et al., 2010) but also to reduce vehicle collisions (Seiler et al., 2004; Seiler & Bhardwaj, 2019).

During the last few decades, the management policy in Scandinavia has therefore generally been to limit moose population density by harvest to levels well below density‐dependent resource limitation (Grøtan et al., 2009; Holmes et al., 2021; Sæther et al., 1996), which also means that the harvest of moose is restricted to levels below the biological potential for maximum sustainable yield. In this system, predation on moose from wolves and brown bears will be largely additive to starvation and diseases as the vast majority of moose killed tend to be in good body condition (Sand, Wikenros, et al., 2012; Swenson et al., 2007). This is further supported by the fact that annual natural mortality of moose in areas without large carnivores is low (<5%) in this population (Broman et al., 2002; Ericsson & Wallin, 2001; Solberg et al., 2018) and that there were significant differences in moose mortality inside versus outside one wolf territory (Gundersen et al., 2008). Therefore, theoretical models of the effect of predation from these two carnivores have in this system predicted significantly reduced opportunities for human harvest (Jonzén et al., 2013; Nilsen et al., 2005). A few years later, Wikenros et al. (2015) showed empirically that harvest on moose in Sweden was significantly reduced following the establishment of wolf territories, as compared to wolf‐free areas. However, the authors argued that this reduction was mainly a result of a functional response among hunters and managers to an anticipated increase in predation mortality in the moose population rather than a direct numerical effect of wolf predation on the moose population. Results also suggested that the reduction in harvest was highly variable both within wolf territories over time and among wolf territories and that hunters in some areas likely overestimated the effect of wolf predation, and therefore reduced harvest more than necessary to compensate for the new mortality factor. In a following study, the authors showed that both the size of total moose harvest and of adult females was strongly negatively related to wolf territory density (Wikenros et al., 2020). Yet another study investigating the impact of wolves and bears on the moose population showed that calf per cow ratios in fall were negatively correlated with wolf and brown bear densities, suggesting that predation was an important factor of summer calf survival (Tallian et al., 2021). However, the extent to which wolves are limiting moose population growth and the potential for harvest has not previously been investigated in this system.

Here, we quantify causes of mortality of a joint resource (moose) between (1) a controversial recolonizing large carnivore (the wolf), (2) an expanding population of another large predator (the brown bear), (3) humans (harvest), and (4) other mortality in a system characterized by strong anthropogenic impact on all trophic levels. We used extensive empirical data on wolf predation on moose collected from studies in 20 wolf territories during multiple seasons and combined these with area‐specific data on moose harvest, brown bear predation, and mortality from vehicle collisions and other causes. We also examined prey‐ and predator‐related factors (moose density, wolf territory size, wolf kill rate, wolf pack size, wolf density, and brown bear density) of importance for wolf predation rates on moose inside the 20 distinct wolf territories. Finally, we tested if and to what extent predation from these two large predator species and human harvest were compensatory to each other at the wolf territory level. Based on the existing literature, we predicted that (1) wolf predation rate should be strongly related to the ratio of moose to wolves within wolf territories and that kill rate per se would be a poor predictor of predation rate (Messier, 1994; Vucetich et al., 2011); (2) human harvest should be the dominating mortality factor on moose within most wolf territories (Jonzén et al., 2013; Nilsen et al., 2005); and (3) local moose populations exposed to relatively high predation rates from wolves and brown bears should be compensated by a management strategy of intentionally reduced harvest to avoid an overall reduction in moose density and future harvest (Wikenros et al., 2015, 2020). Finally, we discuss our results in relation to similar data from other moose populations in the context of variable anthropogenic impact on multiple trophic levels on the ecosystem and the consequences for moose management.

## METHODS

### Study area

**FIGURE 1:**
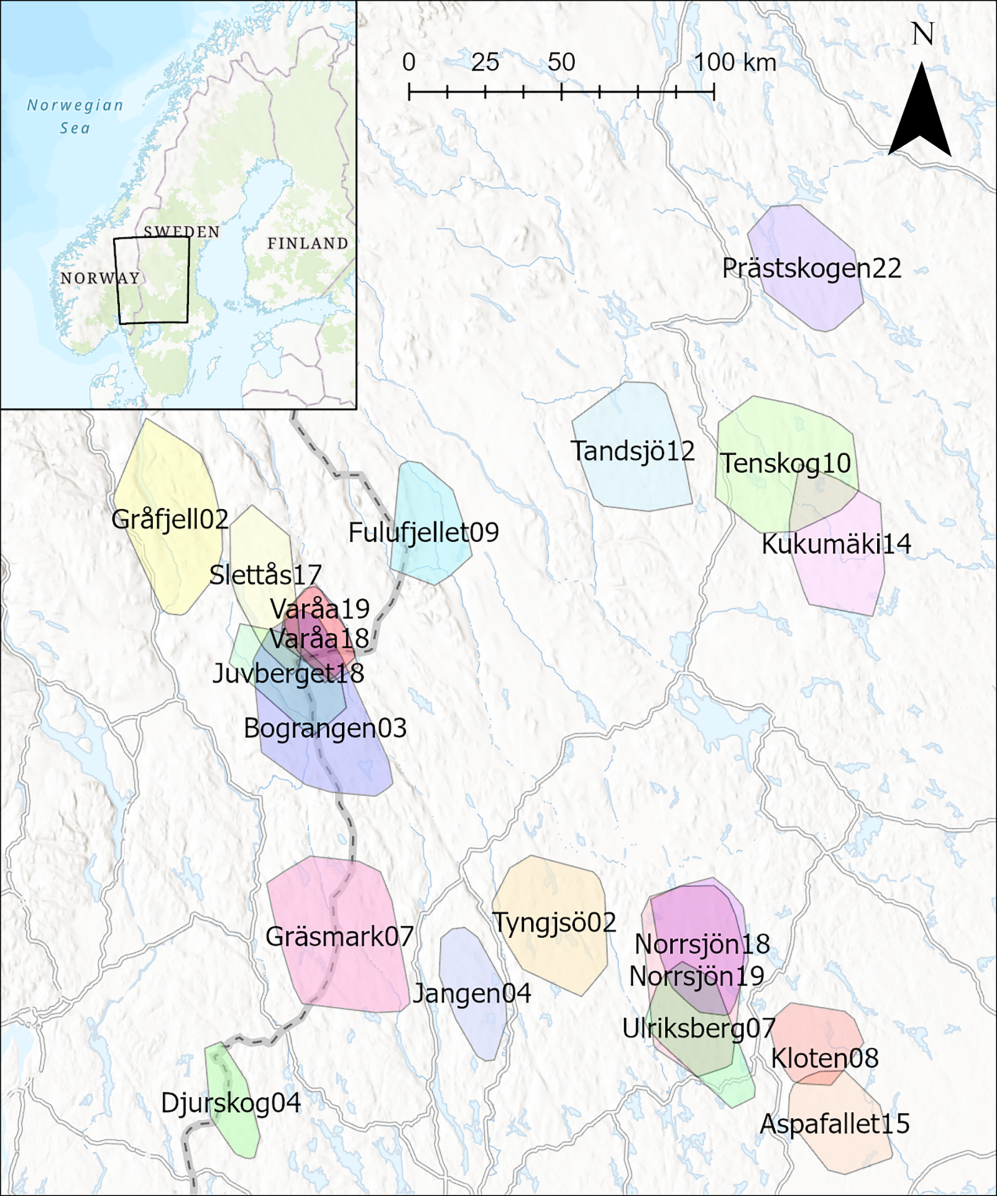
Wolf territories included in the study of wolf and brown bear predation impact in Scandinavia during 2002–2022. Numbers following the territory name indicate the year of study (2002–2022).

The wolf‐breeding range in Scandinavia is limited to the central and southern parts of Sweden and the adjacent areas in southeastern Norway, at 59°–63° North and 11°–19° East (Figure 1). The recolonizing wolf population (Wabakken et al., 2001) has had a positive growth ranging from 106 to 540 wolves during the study period (2002–2022) and with a scattered distribution of territories indicating a non‐saturated population (Rodríguez‐Recio et al., 2022; Wabakken et al., 2002, 2022). Similarly, the brown bear population in Scandinavia has increased in size and range during most of the 20th century (Kindberg et al., 2011; Swenson et al., 1995) with an estimated population size at 2700–3200 bears during 2012–2018 (Bischof et al., 2020). Both wolves and brown bears in central Scandinavia utilize moose as their main prey species (Ordiz et al., 2020; Sand et al., 2005, 2008; Swenson et al., 2007). The continental climate is characterized by cold, dry winters with snow normally covering the ground from November to April (Swedish Meteorological and Hydrological Institute, https://opendata-download-metobs.smhi.se/explore/). The boreal forest zone of this area is dominated by Scots pine, Norway spruce (*Picea abies* L.), and birch (*Betula* spp.), intermixed with a few other deciduous tree species (Roberge et al., 2020). Moose is the dominant wild cervid species with densities ranging from 0.5 to 2.0 moose km^−2^, but we also find low densities of roe deer (*Capreolus capreolus* L.) (<0.1 roe deer km^−2^) in most parts of the study area, and red deer (*Cervus elaphus* L.) in restricted parts of the area (Sand et al., 2016). Even though human population density averages 16 inhabitants km^−2^ throughout Scandinavia, vast areas within the wolf population range have fewer than 1 inhabitant km^−2^ (Statistics Norway, 2018; Statistics Sweden, 2018). Despite relatively low human density, the Scandinavian boreal forest ecosystem can be characterized by having a strong anthropogenic impact on several trophic levels (Kuijper et al., 2016). Forests are intensively managed for timber and pulp whereby mature stands are harvested by clearcutting and then reforested by planting or natural regeneration, resulting in even‐aged coniferous forest stands often mixed with birch (Jansson & Antonson, 2011). In the southern parts of the study area, agriculture is intensive with the proportion of arable land sometimes dominating over forest. The intensive silviculture has led to an extensive forest road network, and road densities, including regional and national roads, average 2.0 km km^−2^ in the study area (NVDB, n.d.). Moose harvest is also intensive throughout with some 25%–30% of the preharvest moose population killed annually in areas without carnivores (Jonzén et al., 2013; Kalén et al., 2022; Nilsen et al., 2005). Similar to moose, the harvest of wolves and brown bears occurs annually with the objective to restrict or slow down further population growth and distribution of the two predator species (Bischof et al., 2020; Liberg et al., 2020).

### Capture of wolves

Within wolf territories, adult wolves and occasionally pups (<1 year old) were collared with VHF collars in 1999–2001 (Telonics), and with GPS devices in 2002–2022 (SimplexTM of Followit AB [Lindesberg, Sweden], or GPS plus of Vectronic [Berlin, Germany]). Animal capture and handling techniques are described by Sand et al. (2006) with a more recent update (Arnemo & Evans, 2017). The size and age structure of studied wolf packs were estimated by ground‐tracking on snow (Wabakken et al., 2001) supported by DNA identification of individual wolves (Åkesson et al., 2022; Liberg, Aronson, et al., 2012). Two scent‐marking adult wolves of opposite sexes without pups occupying a territory are hereafter called pairs, whereas packs of more than two wolves were equal to the reproducing pair and their offspring. Pack size is defined as the maximum number of pack members registered from tracking on snow during winter studies of predation within the wolf territory.

### Studies of wolf predation and estimation of kill rate

During 2002–2022, we estimated wolf territory‐specific kill rates for packs and pairs with winter study periods starting as early as 6 November and ending as late as 24 April and summer study periods starting 1 June and ending 30 September (Table 1). Wolves were monitored with 1‐h (winter) or 30‐min positioning intervals (summer). Positions were retrieved for 92% of all positioning attempts. After biweekly VHF downloading or continuous GSM transferring of GPS data, clusters of positions were searched in the field for wolf‐killed prey. The methods for searching, finding, and classifying killed prey from the GPS data are described in Sand et al. (2005, 2008) and Zimmermann et al. (2007). For periods other than predation studies, we used a standard GPS fix interval that included a minimum of two positions per day. These data together with GPS data from study periods was used to calculate annual minimum convex polygons (100% MCPs) as an estimate of wolf territory size.

**TABLE 1:** Wolf territories included in the study, year, wolf territory size (annual 100% MCP), wolf group size (in winter), the estimated number of moose present within the wolf territory post‐calving (1 June, *M*_start_) and at preharvest (9 October), the number of moose harvested, the estimated annual number of wolf‐killed and brown bear‐killed moose, and the annual number of moose estimated to have been killed by traffic and other causes (starvation, disease, and traffic‐related) within each wolf territory.

Wolf territory	Year of estimate	Wolf territory size	Wolf group size	No. moose 1 Jun	No. moose 9 Oct	No. moose harvested	Wolf predation mortality	Brown bear predation mortality	Traffic and other mortality	Total moose mortality rate (%)
Aspafallet	2015	1162	4	1170	1112	172	81	1	22	23.8
Bograngen	2003	4627	2	4628	4545	921	131	5	29	23.5
Djurskog	2004	1011	5	1013	937	204	137	0	3	34.0
Fulufjället	2009	1241	6	1233	1112	145	109	44	9	24.9
Gräsmark	2007	3905	5.5	3911	3839	766	148	3	37	24.5
Gråfjell	2002	2827	3	2832	2754	386	123	6	34	19.5
Jangen	2004	878	2	884	819	293	110	1	24	48.7
Juvberget	2018	1010	7	1020	943	202	124	5	7	33.2
Kloten	2008	955	2	959	900	78	117	1	7	21.2
Kukumäki	2014	2382	3	2400	2169	200	87	171	17	19.9
Norrsjön	2018	1054	8	1080	1012	255	98	6	17	35.0
Norrsjön	2019	2180	5	2201	2133	482	116	7	20	28.5
Prästskogen	2022	1228	6	1240	1094	160	92	91	7	28.3
Slettås	2017	870	9.5	873	812	184	102	2	7	33.9
Tandsjön	2012	916	5	927	758	163	91	118	3	40.6
Tenskog	2010	1705	2	1721	1451	187	99	203	14	29.3
Tyngsjö	2002	1947	6	1953	1891	225	119	2	9	18.2
Ulriksberg	2007	1161	7	1166	1101	292	101	3	17	35.6
Varåa	2018	442	2	451	395	108	76	2	2	41.7
Varåa	2019	819	2	834	775	105	100	3	9	26.1
Mean		1028	4.6	1625	1527	276	104^a^	34	15	29.5
1.96 × SE		183	1	478	476	97	5	27	5	3.6

*Note*: For variation of wolf predation estimates, see Appendix S1: Table S1.

^a^ Inverse variance‐weighted mean (Appendix S1).

In total, we performed 20 winter studies and 13 summer studies on 18 different wolf packs generating a corresponding number of estimates of kill rate for both seasons (included are two packs studied for two consecutive winters) (Table 1; Appendix S1). Winter study periods ranged from 42 to 132 days (59 ± 9.6 days [mean ± 95% CI]) totaling 1172 days. Summer study periods ranged from 27 to 77 days (41 ± 8.7 days) totaling 538 days. For wolf packs that were not studied during summer (*n* = 7), we applied either the average estimated kill rate from the 13 summer studies (*n* = 5) or, when available, we used the estimated kill rate from the same wolf pack for a different year (*n* = 2).

Zimmermann et al. (2015) found no evidence that pups in our study area killed moose while traveling without their parents within the natal territory. The pups mainly fed on kills made by the adult wolves or on carcasses of unknown causes of death (Zimmermann et al., 2015). Based on these findings, we assumed that the adult wolves were responsible for the vast majority of moose kills, but we acknowledge that in some territories, yearlings without collars may have been present and contributed to additional moose kills, especially during summer (Sand et al., 2008).

To estimate annual moose predation by wolves, we divided the year into two periods consisting of 132 summer days (1 June–9 October) and 223 winter days (10 October–31 May). Previous studies have shown wolf kill rates on moose to differ between these two periods of the year (Sand et al., 2005, 2008; Sand, Vucetich, et al., 2012), and kill rates on moose during summer are strongly related to time (date) as a response to the body growth of juvenile moose, which is their primary prey type during both summer (90% of all moose killed) and winter (70%) (Sand et al., 2005, 2008). Thus, moose calves are highly selected by wolves, as their fraction normally constitutes 25%–35% of the winter population (Sand, Vucetich, et al., 2012).

Based on results from these earlier studies, we estimated territory‐specific total summer predation by applying a linear mixed model, with the square root transformed interval between consecutive kills as the response variable (Sand et al., 2008), Julian date starting at 1 June as a fixed effect, and wolf territory id as a random factor. This way, we assumed that kill intervals increased with time during the summer period at a nonlinear rate for all wolf packs, but that the baseline interval (random intercept) differed among packs (Appendix S1: Figure S1). We then predicted for each day and wolf territory during the summer period the daily kill rate and obtained the territory‐specific summer kill rate by summing the daily kill rates (Appendix S1: Table S1).

Next, we estimated the total wolf predation for the winter period by first modeling the square‐root‐transformed time interval between kills as a function of Julian date (linear mixed model with territory as random factor). Unlike the summer model, the Julian date did not explain the variation in the time interval between kills (*p* = 0.771). We therefore estimated the territory‐specific winter kill rate by estimating daily kill rates from the average time interval between kills found during the winter study period and multiplying these with the length of the winter period (Appendix S1). Total annual wolf predation was calculated as the sum of the territory‐specific summer and winter predation (Table 1; Appendix S1: Table S1).

### Brown bear densities and kill rates on moose

For brown bear densities inside wolf territories (Appendix S1: Table S1), we used two datasets: (1) published annual estimates on brown bear densities from spatial capture‐recapture models (Bischof et al., 2020) based on genetic samples registered in the Scandinavian carnivore registry (www.rovbase.no) for the 2012–2018 period, and (2) annual kernel density maps (ArcGIS Pro 3.0.2, Esri) of harvested bears, based on the locations where bears had been shot (www.rovbase.no). For wolf predation studies during the period 2012–2018, we extracted mean bear density estimates in the wolf territories directly from the first dataset. For predation studies before and after this period, we used dataset 2 to estimate live bear density from the number of harvested bears. We derived a conversion factor by extracting annual densities of both live (dataset 1) and harvested bears (dataset 2) in the years 2012–2018 to all wolf territories and applying a linear model (live bear densities ~ harvested bear densities + year). Assuming that the ratio of live to dead bears was constant in time and space, we used the resulting coefficient of harvested bear density (3.62) as a conversion factor to estimate live bear density. However, only 60% of the bear population is assumed to have attained an age when individuals become independent predators on moose, and consequently, we multiplied all density estimates by 0.6 to obtain estimates for adult brown bear density (J. Kindberg, personal communication). Next, we combined the adult brown bear density estimates with estimates on brown bear kill rates on moose (Ordiz et al., 2020; Rauset et al., 2012; Swenson et al., 2007) to calculate the total annual predation by brown bears within each wolf territory. Because the three studies on brown bear kill rates referred to above gave somewhat different estimates of the total annual number of moose killed per adult bear (6.8 in Swenson et al., 2007: 7.6 in Rauset et al., 2012: 4.5 in Ordiz et al., 2020, in areas overlapping with wolf territories), we used an intermediate estimate of 5 moose per adult brown bear^−1^ year^−1^.

### Estimates of moose population density

To estimate the relative density of moose and other cervids, we carried out fecal pellet group (FPG) counts in each of the 20 wolf territory‐years (Månsson et al., 2011; Neff, 1968; Rönnegård et al., 2008). The sampling design consisted of 38–192 systematically distributed sampling squares of either 1 × 1 km (Appendix S1: Table S1), with 40 circular sample plots along the 4‐km perimeter of each square at a constant distance of 100 m, or sampling squares of 50 × 50 m, with four circular sample plots on each edge and a fifth plot in the center of the square. All FPGs deposited after leaf fall during the previous autumn were counted in each 100‐m^2^ plot shortly after snow melt. To distinguish winter FPGs from older FPGs in the field, we looked at the position of the FPGs in relation to vegetation and leaf litter, and the state of decomposition of the pellets. After leaf fall, the decay rate is very low due to temperatures mostly below 0°C and snow cover for most of the time. We divided the density of FPGs found per plot by the duration of the sampling period, that is, from 10 October to the date of sampling in the following spring, resulting in a daily FPG deposition density, hereafter “FPG density.” The average FPG density per sample square was interpolated across the total pellet count area using inverse distance weighting (ArcGIS Pro 3.02; Esri). From the resulting raster, we estimated the average FPG density within the wolf territory. Wolf territory size was estimated with the 100% MCP method and varied greatly between packs (Table 1) but was not correlated with the duration of the territory‐specific winter study (Spearman's *r*_*s*_ = 0.32, *p* = 0.17, two‐tailed test). We converted the average FPG density into an estimated number of moose for each territory by dividing it by an average daily defecation rate of 14 FPGs per moose. This defecation rate was received from a Scandinavian study area where the population size from aerial moose counts was matched with the number of FPGs in two separate years (14.4 ± 0.71 [mean ± SE] in 2002 and 13.4 ± 0.83 in 2006; Rönnegård et al., 2008).

### Harvest data

In Scandinavia, local moose populations are managed by harvest according to management plans made within geographically defined moose management units (MMUs). MMUs are administrated by the county administrative boards in Sweden and the municipalities in Norway (see Wikenros et al., 2020 for further details). Each MMU consists of a number of smaller management areas of various sizes, for which hunters report the number of harvested moose. These harvest statistics are compiled at the MMU level by the Swedish Association for Hunting and Wildlife Management (www.viltdata.se, Ordiz et al., 2015), the county administrative boards in Sweden (https://www.algdata.se/), the Norwegian municipalities (https://www.hjorteviltregisteret.no/, Norwegian Environment Agency), and Statistics Norway (www.ssb.no). Harvest data per MMU were retrieved from these sources. Annual harvest in each wolf territory was then calculated as the area‐weighted average for those MMUs that overlapped in time and space with a given wolf territory (Table 1).

### Traffic and other mortality

We extracted all registered vehicle‐related accidents with moose, including location and date, from national databases in Sweden for the years 2010–2020 (Nationella Viltolycksrådet, https://www.viltolycka.se/) and in Norway for all years (Hjorteviltregisteret, https://www.hjorteviltregisteret.no/). We assumed that nearly all reported car‐related accidents were lethal for the moose involved (Seiler & Bhardwaj, 2019) and that a small number of non‐reported collisions also were lethal and approximately equated the small number of nonlethal collisions in the database. For wolf territories studied in 2010–2020, we counted the number of collisions within each territory for a given study year. For wolf territories studied before 2010 and after 2020, we applied the mean number of moose‐car collisions found for the 2010–2020 period in the same area, as there was no temporal trend in the number of collisions during this period. We also estimated the number of moose‐train collisions by calculating the total km railway within each wolf territory. From this estimation, we applied an annual train‐moose collision rate of eight moose per 100 km railway (Wikenros et al., 2013) and assumed that all collisions were lethal. We then summed car‐ and train‐related mortality into traffic‐related mortality (Appendix S1: Table S1). Based on previous studies of GPS‐collared moose (Broman et al., 2002; Ericsson & Wallin, 2001), we assumed that all traffic‐related mortality was of approximately the same magnitude as other non‐predation and non‐harvest (e.g., starvation, disease, and accidents) mortality, and we therefore applied this relation to calculate the number of moose dying from other causes of mortality in the local moose population.

### Calculations of moose population size, predation rate, and harvest rate

Because some moose were harvested, wolf‐killed, or died from traffic or other causes of mortality during the accumulation period of FPGs (10 October–31 May), we adjusted the total number of moose estimated from the FPG counts in spring by adding missing animals, adjusted for when they might have died during the winter period (Appendix S2). Because a fraction (19% of the calves and 7% of the adults) of the moose killed by wolves during winter previously has been found to be compensatory to other mortality, that is, would likely have died of starvation if they were not killed by wolves, we adjusted the size of other mortality to account for this compensatory mortality source (Sand, Wikenros, et al., 2012; Appendix S2). On top of the estimated number of moose on 10 October, we added all animals that had died in the period 1 June–9 October. This resulted in an estimate of moose abundance in wolf territories by 1 June (*M*_start_), right after assumed calving (Nicholson et al., 2019), and, when divided by the territory area, an estimate of the initial moose density on 1 June (Appendix S2).

We then calculated the total annual harvest and predation rates for each wolf territory and study year (1 June–31 May) by dividing estimated annual harvest and predation from wolves and brown bears by the number of moose at *M*_start_. Total annual mortality in each wolf territory was then calculated as the sum of the rates of harvest, wolf predation, bear predation, traffic‐related mortality, and other mortality. The number of years since wolf territory establishment for specific wolf territories was derived from reports of the annual monitoring of the wolf population (e.g., Wabakken et al., 2002, 2022).

### Analyses

To assess potentially important predictors of the observed variation in wolf predation rates between wolf territories, we performed multiple univariate regressions including the following variables: territory size, pack size, wolf density (pack size/territory size), the total abundance of moose within the wolf territory *M*_start_, moose density (*M*_start_/territory size), the ratio of moose to wolves, and the total annual predation by wolves. We tested for both linear, logarithmic, exponential, and quadratic relationships between the response (predation rate) and explanatory variables (listed above) using the nls2 package in R version 4.1.3 (R Core Team, 2022). We used the same procedure for moose harvest rate but tested for correlation with only two meaningful variables: wolf predation rate and the combined predation rate from wolves and brown bears. To identify top models among the linear and nonlinear univariate models, we used corrected Akaike information criterion (AIC_c_) model selection where models with ΔAIC_c_ < 2 were considered equally good (Appendix S3). We calculated an *R* ^2^ value that described the proportion of the variation in the response variable that could be explained by the model. For nonlinear models this parameter can be calculated as

(1) $$R_{i}^{2}=1-\frac{{\mathrm{SSE}}_{i}}{{\mathrm{SST}}_{\text{Null}}},$$

where SSE_*i*_ is the squared sum of the residuals for a given model and SST_Null_ is the total squared sum of the null model (Spiess & Neumeyer, 2010). In addition, we calculated the *p* value from an ANOVA comparison of a given model *i* with the null model (Appendix S3: Tables S1 and S2).

Finally, we performed a generalized linear model for proportional data with a quasi‐binomial link to test whether the observed variation in the wolf predation rate across territories could be explained by a combined effect of moose and wolf density. These two predictors are relevant from a management perspective, because both moose and wolf population densities are subject to human regulation by harvest. We used McFadden's *R* ^2^ (1 − deviance/null deviance) to estimate the proportion of explained variation. Analyses are presented according to the ESA Guidelines for Statistical Analysis.

## RESULTS

### Wolf population characteristics and moose abundance

Wolf territory size (annual 100% MCP) averaged 1028 ± 187 km^2^ (mean ± 95% CI, range = 420–1823 km^2^, *n* = 20) (Table 1). Average pack size was 4.6 ± 1.0 (range = 2–10) wolves resulting in an average wolf density within territories of 5.0 ± 1.3 wolves/1000 km^2^ (range = 1.2–11.9). An estimation of the total abundance of moose within wolf territories at the start of the study year post‐calving (*M*_start_ = 1 June) averaged 1625 ± 478 individuals (range = 451–4628), which was equal to an average density of 1.58 ± 0.33 moose/km^2^ (range = 0.75–4.20). The estimated abundance of moose before the onset of moose harvest (set to 10 October) averaged 1527 ± 476 moose (range = 395–4545; Table 1) and in spring before calving (set to 31 May) the following year 1190 ± 384 moose (range = 263–3538). Wolf territory size was neither correlated with pack size (Spearman's *r*_*S*_ = 0.21, *p* = 0.38) nor with the estimated moose density on 1 June (*r*_*S*_ = 0.06, *p* = 0.79).

### Predation, harvest, and mortality

Average total annual wolf predation was estimated to be 104 ± 5 (range = 76–148) moose per wolf territory (Appendix S1: Table S1). An estimation of annual brown bear predation on moose ranged from 0 to 203 moose killed per wolf territory, with a median of 4 moose. This right‐skewed distribution of bear kill rate was a result of the clumped distribution of bears in the wolf population range, with estimated bear densities ranging from 0 to 48 bears/1000 km^2^ (median = 1.5). Average estimated annual number of moose harvested was 276 ± 95 (range = 78–921) (Table 1). Finally, the estimated annual number of moose killed by traffic and other mortality together accounted for an average of 15 ± 5 (range = 2–37) moose per wolf territory.

**FIGURE 2:**
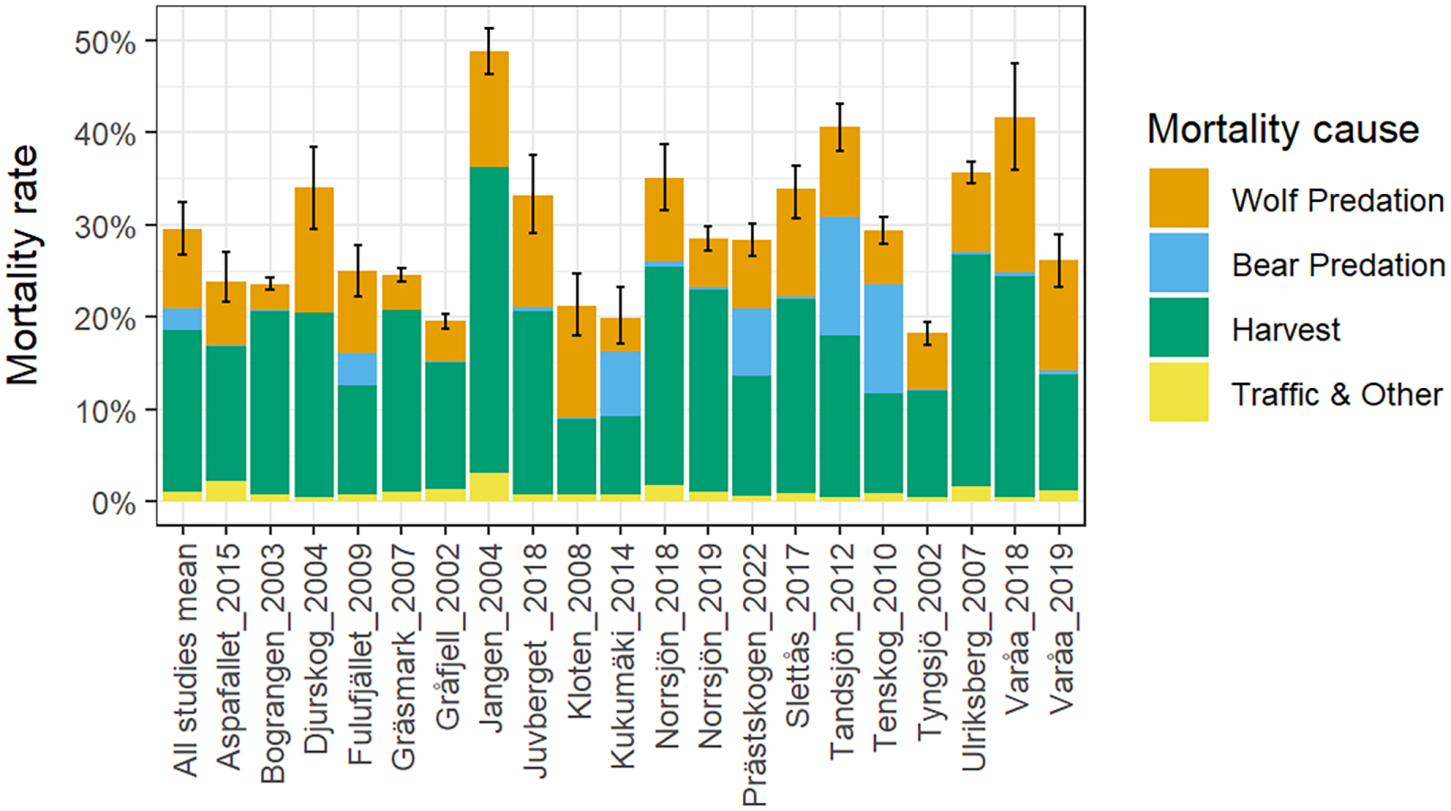
Estimated annual mortality of moose in 20 wolf territories in Scandinavia during 2002–2022, divided into predation rate by wolves and brown bears, harvest rate from hunters, and traffic‐related and other mortality. Mean total mortality across all wolf territories is given in the left‐most bar. For wolf predation rate, the territory‐specific 95% CIs are indicated with error bars.

Wolf annual predation rate, estimated as the proportion of the post‐calving moose population (*M*_start_) killed by wolves, was on average 8.7% ± 1.7% (range = 2.8%–16.9%) across territories (Figure 2). Corresponding estimates for brown bear predation and human harvest were 2.3% ± 1.8% (mean ± 95% CI, range = 0–12.7) and 17.5% ± 2.8% (range = 8.1–33.1) respectively, whereas traffic and other mortality together accounted for 1.1% ± 0.3% (range = 0.4–3.0) (Figure 2). Thus, the total annual moose mortality rate within wolf territories was on average 29.5% ± 3.6% and ranged 18.2%–48.7% among territories.

The proportion of the total annual moose mortality caused by wolf predation among the 20 wolf territories was estimated to be 29.2% ± 4.9% (range = 12.0%–57.4%), whereas brown bear predation and harvest accounted for 8.0% ± 6.0% (range = 0%–40.3%) and 59.1% ± 6.1% (range = 37.1%–84.5%), respectively. Harvest was thereby, on average, 2.4 ± 0.7 (range = 0.67–7.03) times higher than wolf predation among all 20 wolf territories. Restricting the same type of comparison for brown bear predation to the four territories with the highest densities of brown bears (Kukumäki, Tandsjön, Tenskog, Prästskogen; 29–48 bears/1000 km^2^) showed that harvest was only 1.3 ± 0.3 (range = 0.9–1.8) times higher than brown bear predation. In the same four wolf territories, brown bear predation was 1.6 ± 0.5 (range = 1.0–2.1) times higher than wolf predation. In only one territory (Kloten) was wolf predation higher than harvest mortality and in five additional wolf territories (Fulufjället, Kukumäki, Tandsjön, Tenskog, and Prästskogen) was the combined predation from wolves and brown bears higher than harvest (Figure 2). Across all wolf territories, harvest was on average 2.1 ± 0.7 (range = 0.6–6.8) times higher than wolf and brown bear predation combined.

### Factors affecting wolf predation rate among wolf territories

**FIGURE 3:**
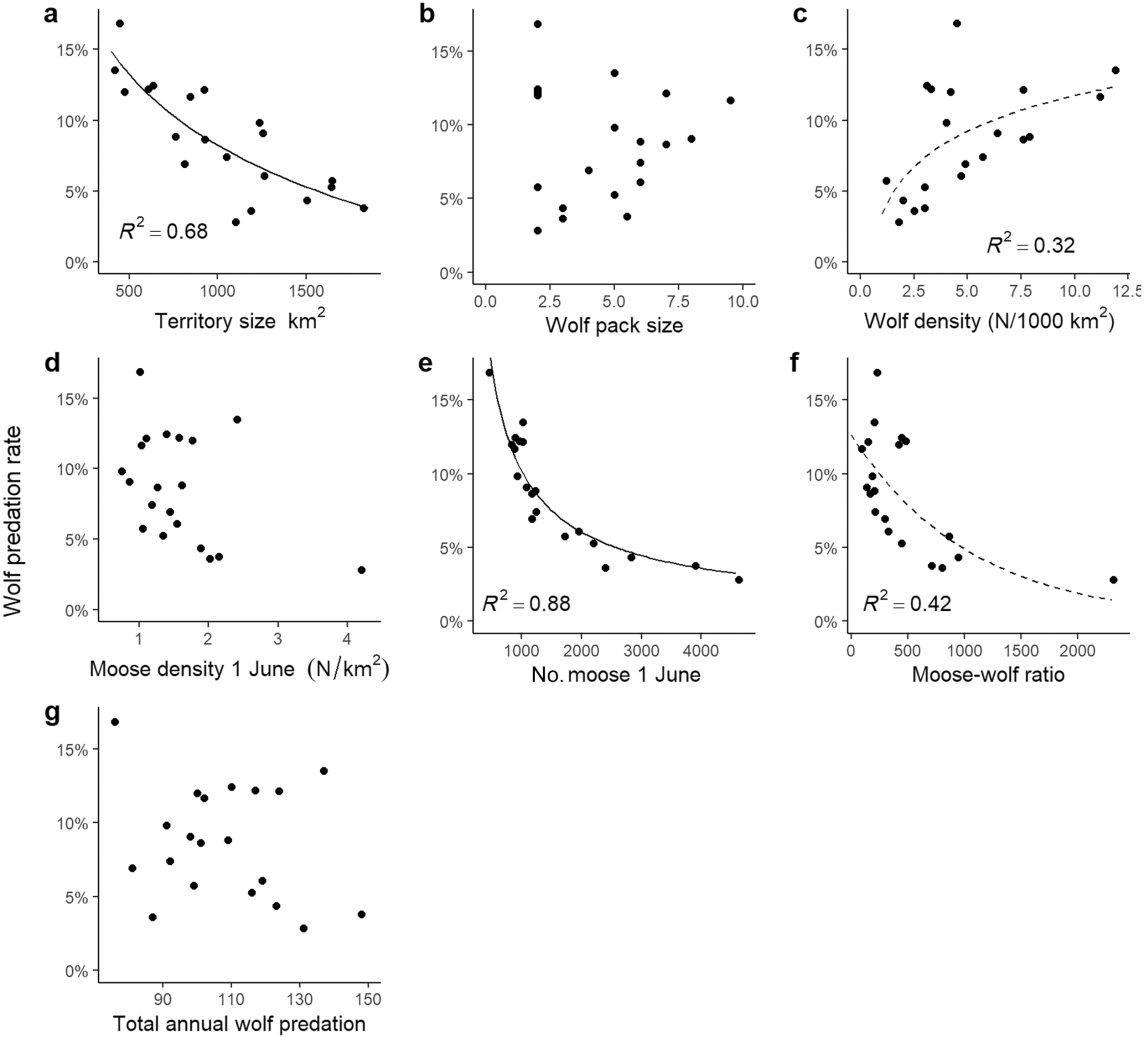
The annual proportion of the moose population after calving in June which was killed by wolves until 31 May the following year relative to (a) territory size, (b) wolf pack size, (c) wolf density, (d) moose density by 1 June, (e) number of moose in the territory by 1 June, (f) moose–wolf ratio, and (g) total annual wolf predation in Scandinavia. Trend lines are included where the null model (assuming no relationship between predation rate and the explanatory variables) was not among the top models (solid line) or was not the only top model (dashed line, difference in corrected Akaike information criterion between models [ΔAIC_c_] < 2).

An analysis of the relationship between wolf predation rate and single wolf‐ and moose‐related explanatory variables showed a strong relationship between wolf predation rate and wolf territory size (Figure 3a). The relationship was best explained with a negative logarithmic function (predation rate = 0.5813–0.07223 × log(territory size)). For wolf territories of 500, 1000, and 1500 km^2^, this function predicted a wolf predation rate of 13.2%, 8.2%, and 5.3%, respectively. Wolf predation rate was not related to pack size (Figure 3b), but weakly to wolf density (Figure 3c). Although wolf density did not give a lower AIC_c_ than the null model, the logarithmic model (*R* ^2^ = 32%) was within ΔAIC_c_ < 2, and all linear and nonlinear regressions had a better fit (smaller sum of squared residuals) than the null model (Appendix S3: Table S1). The logarithmic model (predation rate = 0.03418 + 0.03621 × log(wolf density)) predicted a predation rate of 5.9%, 9.9%, and 12.4% for 2, 6, and 12 wolves/1000 km^2^, respectively.

We could not find any relation between the estimates of wolf predation rate and moose population density on 1 June (Figure 3d). The removal of an outlier (moose density >4 moose/km^2^; Figure 3d) did not improve the model. The combination of wolf territory size and moose density determined the estimated total number of moose available to wolves within their territory. In fact, 88% of the variation in the estimated wolf predation rate between wolf territories could be attributed to the total number of moose available to wolves on 1 June (negative power function, predation rate = 18.1734 × *M*_start_^−0.7501^; Figure 3e). The relationship between wolf predation rate and the moose‐per‐wolf ratio was also negative (predation rate = 0.1262 × e^−0.00095×moose:wolf ratio^), but not as strong (*R* ^2^ = 42%; Figure 3f). The removal of an outlier (>2000 moose per wolf) resulted in a slightly weaker relationship between predation rate and moose‐per‐wolf ratio (*R* ^2^ = 37%). In contrast, wolf predation rate was not related to variation in the estimated total annual kill between wolf territories (Figure 3g).

**FIGURE 4:**
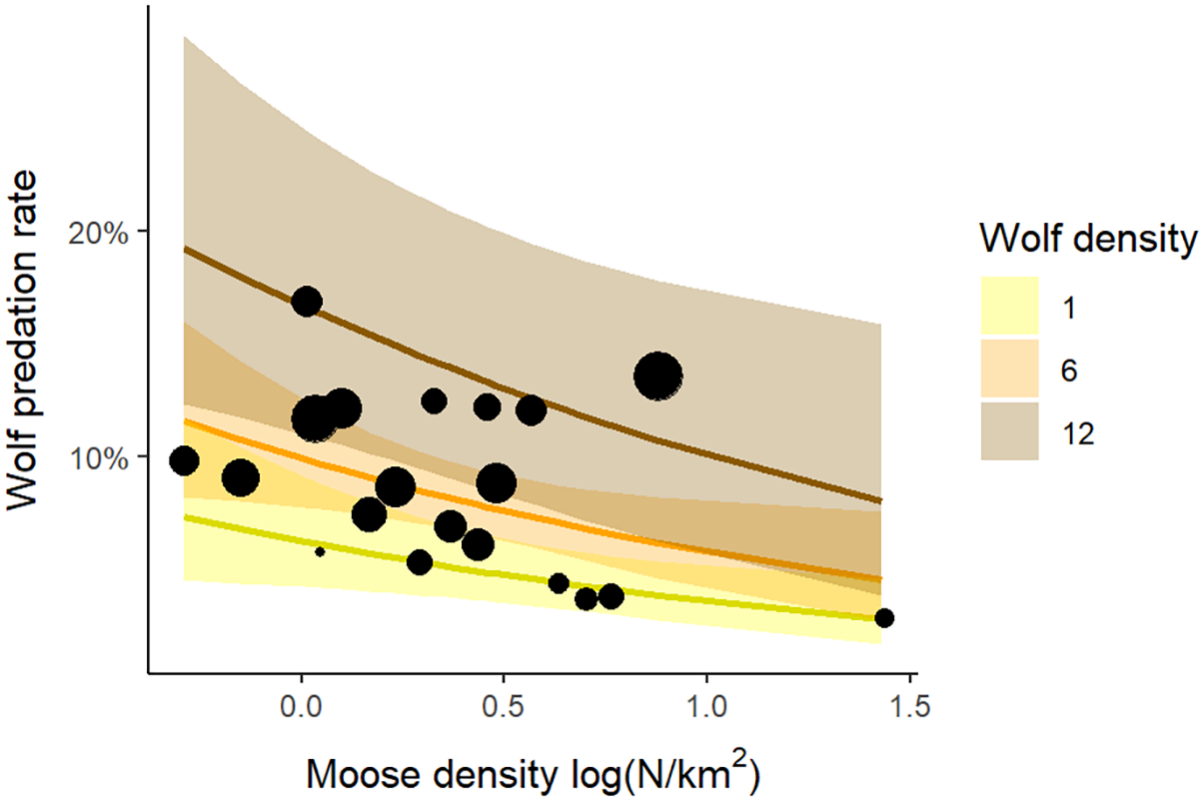
Predicted wolf predation rate on moose (mean and 95% CI) in Scandinavia from a logistic regression model that included both wolf density and the log‐transformed moose density as explanatory variables. The dots represent the studies (*N* = 20), with dot size proportional to wolf density.

Combining moose and wolf density as predictors in a generalized linear model revealed that the wolf predation rate was negatively related to moose density (log‐transformed to account for a high‐density outlier, *p* = 0.019), but positively to wolf density (*p* = 0.005; Figure 4). The model explained 63% of the observed variation in wolf predation rate, compared with 38% and 45% when including moose density and wolf density separately, respectively.

### Harvest rate, predation rate, and total mortality among wolf territories

**FIGURE 5:**
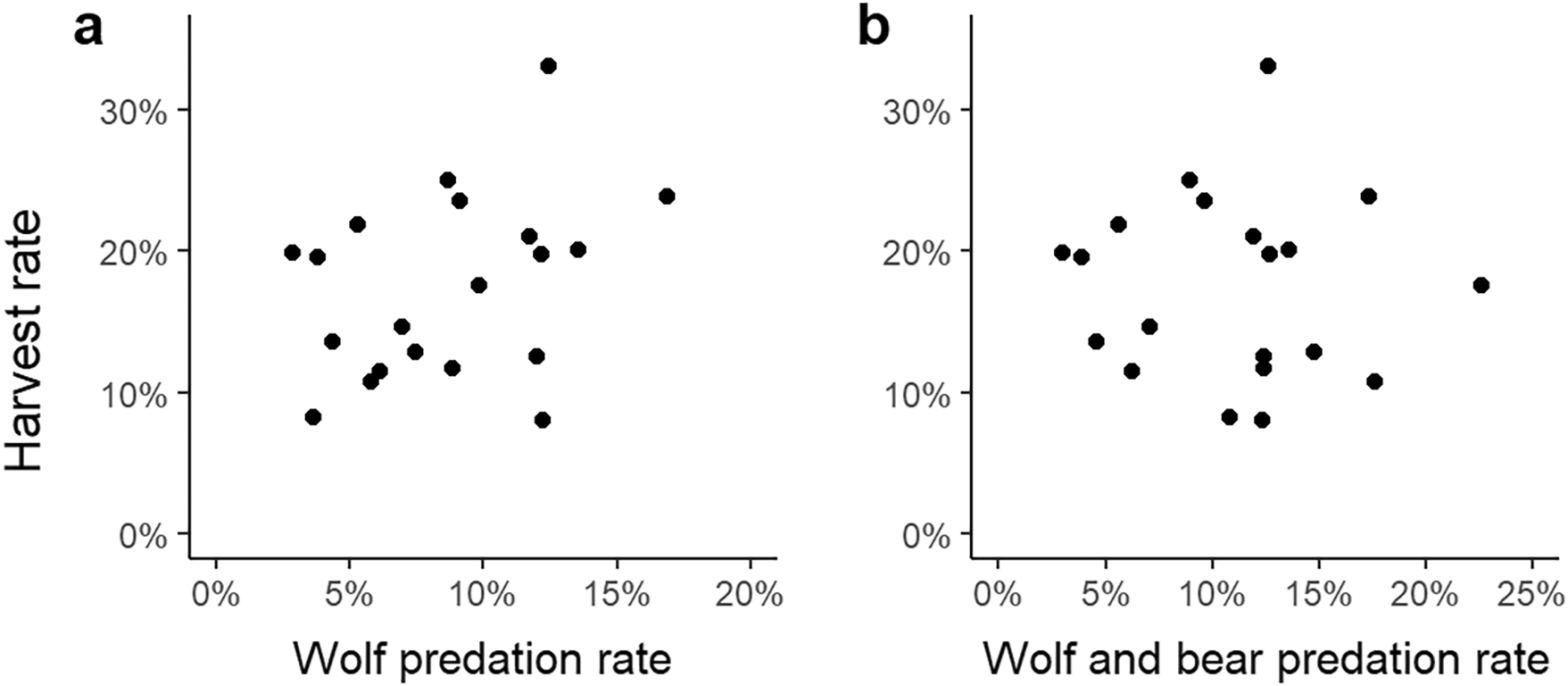
Harvest rate measured as the annual proportion of the moose population after calving in June plotted against (a) wolf predation rate and (b) the combined predation rate by wolves and brown bears in Scandinavia.

As expected, the size of the annual harvest during fall was strongly positively correlated with the estimated number of moose present 1 June (*r*_*s*_ = 0.90). However, the harvest rate was not related to the estimated wolf predation rate nor to the combined estimated predation rate from wolves and brown bears (Figure 5a,b; Appendix S3: Table S2). Since we predicted a negative (compensatory) relationship between the harvest rate and the wolf predation rate, we also tested if harvest rates may have been adjusted to wolf presence in the years previous to the year of study by including a variable representing the number of years since wolf territory establishment in the area (mean = 6.5, range = 1–19). This analysis did not improve the relationship between the harvest rate and the wolf predation rate (generalized linear model for proportional data, wolf predation rate *p* = 0.550, years since wolf establishment *p* = 0.418).

## DISCUSSION

Overall, we found support for our two first predictions but not for our third. Wolf predation rate was related to the ratio of moose to wolves within wolf territories but not to kill rate (Prediction 1). The estimated harvest rates from our models were on average 2.1 times higher than the estimated combined predation rate by wolves and brown bears, but this ratio was largely variable between wolf territories. Thus, despite the increasing populations of wolves and brown bears in Scandinavia, moose populations overlapping with established wolf territories were mainly regulated by harvest and, to a lesser extent, limited by wolf and brown bear predation (Prediction 2). However, harvest rates did not relate to predation rates from wolves and brown bears (Prediction 3).

### What factors affect our estimates of the wolf predation rate?

In our system, wolf predation rate was associated with three variables that all depend on wolf territory size: moose abundance, wolf density, and the moose‐to‐wolf ratio. Moose abundance results from the combination of wolf territory size and moose density and is approximately equivalent to the predator–prey ratio that has been shown to be an important parameter for the predation impact of wolves on prey populations (Jedrzejewski et al., 2002; Vucetich et al., 2002, 2011). This might explain why moose density per se was not associated with wolf predation rate in our study, and only weakly when combined with wolf density.

Because wolves actively defend territories with little spatial overlap and the proportion of wolves in the winter population not belonging to territorial packs is relatively small (<15%) (Chapron et al., 2016; Mech & Boitani, 2003), territory size and, within that, wolf density rather than wolf pack size of a given territory determine the rate of predation on moose in this system. This is because (1) Scandinavian wolf packs have a relatively simple structure mainly consisting of the two adult reproducing individuals and their pups from their last reproduction, sometimes accompanied by yearling offspring (Chapron et al., 2016; Nordli et al., 2023), (2) the two adult reproducing wolves are the ones mainly responsible for the hunting and killing of moose (Zimmermann et al., 2015), and (3) there is no significant relationship between the kill rate on moose and total pack size nor with wolf density (this study). Thus, wolf territory size and any factor that may affect this parameter will be important for the impact of wolves on the moose population in our system.

As this system is characterized by relatively low overall wolf density due to both legal and illegal harvest (Liberg et al., 2020; Liberg, Chapron, et al., 2012) resulting in scattered occurrences of wolf territories, a continued increase in wolf population growth is likely to first result in new wolf territories filling the gaps between territories, followed by a reduction in wolf territory sizes as a direct effect of intensified intraspecific competition among bordering wolf territories (Hayes & Harestad, 2000; Kittle et al., 2015; Rich et al., 2012; Sells et al., 2021). An analysis of the temporal variation in wolf territory sizes in Scandinavia for the 1998–2011 period did not provide any evidence for density‐dependent effects on space use nor was there a significant relationship between wolf territory size and moose density (Mattisson et al., 2013). However, two studies have compared variation in wolf territory sizes among geographically distinct wolf populations in relation to the estimated biomass of ungulate prey. These analyses showed that (1) wolf territory size seems to be largely plastic also among wolf populations where moose constitute the main prey species (Fuller et al., 2003) and (2) average wolf territory size in Scandinavia was much larger than in North America even at corresponding levels of prey biomass (Mattisson et al., 2013). The latter observation was likely attributed to the fact that the Scandinavian wolf population was in a recolonizing stage with a relatively low population size (70–300) and density (~1–3 wolves/1000 km^2^) for the period of study in relation to the available amount of ungulate prey biomass (Mattisson et al., 2013; Rodríguez‐Recio et al., 2022).

The variation in wolf predation rate on moose between territories was not explained by the variation found in kill rate (total annual predation [Prediction 1]). This finding is consistent with the conclusions by Vucetich et al. (2011) who tested the theoretically based prediction that kill rate should be closely related to predation rate. In fact, their study showed that kill rate was a poor predictor of predation rate.

### Other mortality in the moose population

A number of studies in Scandinavia have previously estimated the annual fraction of other mortality (including starvation, diseases, and any other type of lethal accidents) in moose populations (Broman et al., 2002; Ericsson & Wallin, 2001; Sæther et al., 1996; Solberg et al., 2018; Stubsjøen et al., 2000), and results show consistently relatively low estimates but with some variation (2%–7%). Our estimate of traffic‐related and other mortality together accounted for an average of 3.3% of the total annual mortality and 1.1% of mortality of the post‐calving population on 1 June. A previous study focusing on quantifying the extent of compensatory mortality for moose killed by wolves showed that 15% (19% of the calves and 7% of the adults) of the moose killed by wolves during winter had a body condition indicating that they likely would not have survived the winter if not killed by wolves, whereas the corresponding figures for harvested moose were 3.3% and 0% (Sand, Wikenros, et al., 2012). A similar analysis of wolf‐killed moose during the summer period, constituting 56% of estimated total annual wolf‐caused mortality, and bear‐killed moose has not been performed but would likely yield lower estimates of non‐harvest/predation mortality due to a higher availability of high‐quality food as compared to winter conditions (Holmes et al., 2021). However, a small fraction of the moose killed by wolves and brown bears during summer would likely have died from traffic‐related or other mortality. This means that we may have slightly overestimated the additive effect of wolf and brown bear predation, and therefore also the total mortality of moose resulting from the combined effect of wolf and brown bear predation.

### Methods to quantify predation and associated uncertainties

The impact of wolf predation on moose populations has been intensively studied in different ecosystems (Boertje et al., 2010; Clark & Hebblewhite, 2021; Gasaway et al., 1992; Hayes & Harestad, 2000; Jonzén et al., 2013; Messier, 1994; NRC, 1997; Vucetich et al., 2011), and the findings have suggested an impact ranging from small or absent to significant (Ballard et al., 1987; Boertje et al., 2009; Boutin, 1992; Clark & Hebblewhite, 2021). Most of these studies have either used (1) experimental removal of carnivores (Gasaway et al., 1992; Hayes & Harestad, 2000; Testa, 2004), (2) data on kill rates extrapolated to some period of the year (Jedrzejewski et al., 2002; Zimmermann, 2014), (3) data on cause‐specific mortality of a sample of moose individuals with the aid of different types of tracking devices (Ballard et al., 1991; Boertje et al., 2009; Gundersen et al., 2008), or (4) long‐term observational studies of moose and wolves (Peterson et al., 1984; Vucetich et al., 2011).

In Scandinavia, Gundersen et al. (2008) compared three methods to estimate wolf predation rate on moose calves in one wolf territory. These methods included either differences in calf/cow ratios from moose observations made by hunters, or from aerial counts, or data on differences in the loss of moose calves to radio‐collared females inside and outside the wolf territory. Results showed that although their estimated predation rates varied considerably both between years as well as among methods used, moose calf survival within the wolf territory was significantly lower than outside and they concluded that this difference essentially constituted the additive component of wolf predation. Another study in the same region found that annual and winter calf survival rates were 20%–40% lower in the wolf territory compared with previous estimates of moose calf survival in similar areas that lacked wolves (Sivertsen et al., 2012).

In contrast, we used empirically derived data on wolf kill rates from both summer and winter to estimate the total annual predation by wolves and combined this information with empirical estimates of the total harvest, brown bear predation, and other types of mortality for each wolf territory. A potential weakness of our estimates of the wolf predation rate may be that we assumed that our study periods during the winter were approximately representative of the total winter period. Study periods for different wolf territories also had some variation in their timing but were mainly centered on the midwinter (January–March) period. Among the summer predation studies used to model declining kill rates from June to September, data from early summer were overrepresented. Further, despite that we used data from empirical studies of wolves, bears, and moose, we also had to make a number of assumptions in our models of predation rate related to both our estimates of wolf and bear predation, moose population size, and the extent of non‐predation mortality of moose. Thus, sampling effects combined with potential variation in kill rates outside our study periods and the validity of the assumptions made for several other parameters contribute to uncertainty in our estimates of the size and additive nature of the annual predation of wolves and brown bears on moose.

### Age composition of prey

In this study, we incorporated territory‐specific estimates of kill rates on moose and prey and predator densities and limited our study to areas where alternative prey species (to moose) were at low density and therefore not important as prey for wolves and brown bears. Despite this, we could not fully estimate the relative effects of wolf and brown bear predation and human harvest on moose population growth. To do that, we would need to include the effects of age‐ and sex‐related prey selection in our analyses, which is known to strongly impact the demography of prey populations (Caughley, 1977; Gaillard et al., 2000). However, Gervasi et al. (2012) modeled the effects of several predator–prey relationships in Scandinavia including the effects of human harvest, wolf, and brown bear predation on moose and showed that predation rate may be a poor predictor of the overall demographic impact of predation on the moose population. Especially, the age composition of moose emerged as an important underlying predictor of the overall demographic impact of predation. By considering the variation in age composition of moose killed by wolves and hunters in a perturbation analysis, Gervasi et al. (2012) showed that harvest had approximately a three times larger impact as compared to wolf predation as measured by elasticity values of lambda. This is because moose harvested by humans in Scandinavia normally contains a 2–3 times higher proportion of adults (~50%) as compared to moose killed by wolves (~20%) and five times larger as compared to brown bears (10%) (Gervasi et al., 2012; Jonzén et al., 2013; Nilsen et al., 2005). This means that by simply comparing the quantitative estimates of predation rates, as in the current study, we largely underestimate the true relative effect of human harvest as compared to wolf and brown bear predation on moose population growth.

Nevertheless, two previous studies (Jonzén et al., 2013; Nilsen et al., 2005) modeled the effect of wolf predation on the moose population in Scandinavia. Both studies predicted that the addition of wolves into this ecosystem would lead to a significant reduction in harvest yield but that the general relationship between the age and sex structure in harvest and total yield would not be affected. Jonzén et al. (2013) showed that the annual growth rate (lambda) in an average Scandinavian moose population was 24% in the absence of both wolf predation and harvest. The same study also showed that the harvest yield in terms of the number of animals harvested needs to be reduced by an average of 35% in an average‐sized wolf territory at an average winter density of 1.0 moose km^−2^ to fully compensate for wolf predation. Alternatively, moose density must increase by 0.4 moose km^−2^ or the harvest ratio of adult female/adult male needs to be reduced from 50:50 to 38:62 (applying 50% calves in harvest) to fully compensate for the increased mortality due to wolf predation. Interestingly, our empirical estimate from the current study is very similar to the modeled impact of wolves on the moose population with estimated predation rates by wolves being on average 33% of the combined mortality from wolf predation and harvest.

### Estimates of harvest rates

Several previous studies of moose population dynamics in Scandinavia have shown that human harvest constitutes the main regulating factor in areas without wolves (Rönnegård et al., 2008; Solberg et al., 1999; Ueno et al., 2014). The current study shows that harvest is also the most important mortality factor in most local moose populations that overlap with established wolf territories (Prediction 2). However, our results also revealed large variation (range = 8.1%–33.1%) in the estimated harvest rates on moose within wolf territories. We predicted (Prediction 3) that this variation in harvest rate would be negatively related to wolf predation rate or to the combined predation rate from wolves and brown bears. This prediction was based on a previous finding that hunters in Scandinavia responded to the establishment of wolf territories by reducing their harvest of moose (Wikenros et al., 2015), as has also been shown for elk populations in North America (Brodie et al., 2013). Our results failed to support this prediction. This lack of negative relationship between predation and harvest rate among local moose populations may have several but not mutually exclusive explanations: (1) hunters may have compensated for the increased mortality by adjusting the structure of harvest toward a lower fraction of adult females (Wikenros et al., 2015, 2020); (2) moose population productivity may differ geographically due to environmental variation and resource competition (Grøtan et al., 2009); (3) moose management goals may differ between local MMUs (Wikenros et al., 2020); and (4) there are differences between wolf territories in the pattern and extent of seasonal migration of moose (Allen et al., 2016). In summary, these potential mechanisms all suggest that there may not exist, or at least that it may be difficult to identify, any clear causal link between wolf and brown bear predation rate, harvest rate, and population growth among local moose populations in Scandinavia.

### Our results in an international perspective

Boertje et al. (2010) performed an extensive review of 74 studies on the impact of wolf and bear predation on moose in Alaska. Of these 74 studies, 10 focused on examining the importance of predation for mortality in areas with both wolves and brown bears. In 9 of the 10 studies, predation was shown to be the most important factor affecting the dynamics in the moose population compared with other types of mortality such as harvest, starvation, disease, or weather‐related mortality. In the one remaining study, predation and density‐dependent factors were equally important. In 4 of the 10 studies, researchers examined and quantified various mortality factors in the moose population during the year, and in 3 of the 4 studies, moose were at relatively low density (<0.4 moose km^−2^). Predation from bears and wolves was estimated to amount to 31%–41% of the post‐calving population with an additional 2%–6% human harvest (Boertje et al., 2009). This was interpreted by the authors as the mortality rate being too high to allow for positive population growth. As in our study, the researchers concluded that most of the predation mortality was additive to other types of mortality. In areas where wolves were the main predator, a significant reduction in wolf population size usually resulted in a corresponding increase in the moose population. In all four studies, moose calves constituted 75%–80% of all moose killed, a finding almost identical to results from Scandinavia (Sand et al., 2005, 2008; Sand, Vucetich, et al., 2012).

For comparison with the estimates of total predation by bears and wolves on moose in the four populations in Alaska, our average estimates of the combined predation from wolves and brown bears and harvest from Scandinavia amounted to 29.5% of the post‐calving moose population. However, in only four of the wolf territories studied in Scandinavia were the moose also exposed to moderate to high predation from brown bears since these overlapped with medium‐to‐high (29–48 bears/1000 km^−2^) bear densities. In these four wolf territories, the impact of bear predation, that is, the annual predation rate by bears, was on average 1.5 times larger than that from wolves (Rauset et al., 2012; Swenson et al., 2007; Tallian et al., 2017) and the total annual predation rate from both wolves and brown bears was 1.6 times higher (16%) compared with the remaining 16 wolf territories with zero or low adult bear density (10%). The results from the current and the Alaskan studies suggest that moose populations in Alaska would be able to sustain a higher annual mortality rate from harvest and predation than the one in Scandinavia, despite the Scandinavian moose population having been classified as one of the most productive in the world (Cederlund & Markgren, 1989; Lavsund et al., 2003). Although some of the differences in mortality estimates between the Alaskan and the Scandinavian moose populations may be due to observation error in the input parameters, there are two major differences between these two systems that are more likely to explain the observed variation in the total mortality rate. The first is that the two predator populations in Scandinavia are strongly limited by harvest, thus preventing a numerical response to prey density and resulting in a lower predator density as compared to Alaska. The Alaskan studies generally had 2–20 times higher wolf‐moose ratios than currently found in Scandinavia (Sand, Vucetich, et al., 2012, this study). The second, and likely most important difference, is that the harvest rate on moose constitutes a much larger fraction accounting for 17.5% in Scandinavia as compared to an estimated 2%–6% in Alaska (Boertje et al., 2009). In addition, the higher relative harvest in Scandinavia includes a high proportion (40%–60%) of adult (≥1.5 year) moose, of which approximately half are females (Nilsen & Solberg, 2006; Rönnegård et al., 2008; Wikenros et al., 2020). Because the fraction of adult moose that dies annually has a strong impact on the productivity of the moose population (Gervasi et al., 2012, Jonzén et al., 2013), high adult harvest mortality will strongly limit the potential for a moose population to sustain a certain level of mortality. Provided that human harvest in Scandinavia also was directed mainly toward calves and adult male moose, local populations would be able to sustain a total mortality of >40% without resulting in a negative population growth (Jonzén et al., 2013, Nilsen et al., 2005).

### Implications for the functional ecology of the system and management

This study provides novel information and perspectives to the general picture of predator–prey interactions among large carnivores and ungulate prey. By simultaneously studying multiple causes of death, we quantified the relative contribution of predation, harvest, traffic, and other causes to total moose mortality and avoided the pitfall of overestimating the role of predators in single‐cause mortality studies (Vollset et al., 2023). Our study system in Scandinavia is characterized by a strong anthropogenic top‐down impact on several trophic levels, and both predator and prey species are exposed to an intensive management regime, mainly through a strict quota‐based harvest. In this system, human impact is the major structuring factor at all three (vegetation, herbivores, and predators) trophic levels (Kuijper et al., 2016) and dominates moose mortality within most existing wolf territories as shown in this study. Legal harvest and poaching currently control wolf population growth (Liberg, Chapron, et al., 2012; Liberg et al., 2020) and effectively preclude any numerical response that would otherwise be expected in systems with a similar abundance of prey but with less anthropogenic control. Unless future political decisions result in a significant change of either moose (lower) or wolf and brown bear (higher) densities, the functional ecological role of these predators on lower trophic levels in this system will continue to be secondary to the role of humans. However, political decision‐makers are currently under strong pressure from powerful landowners to significantly reduce moose density through increased hunting quotas as motivated by “unacceptably high” moose browsing damages to economically valuable young trees (pine, Pfeffer et al., 2021). Since moose population density is one of the most important driving factors for sustainable harvest size in this system (Jonzén et al., 2013, this study), such a change will sharply accentuate the competitive role of large carnivore predation for moose harvest in the future. This alone, or in combination with further increases in predator densities, is not likely to increase acceptance for wolves in this system and will possibly promote even higher rates of illegal killing (Liberg, Chapron, et al., 2012; Liberg et al., 2020). Our results from this study are currently being integrated into the adaptive management of moose populations in Scandinavia and are providing local MMUs with estimates of predation that can be included in harvest models used for moose.

## AUTHOR CONTRIBUTIONS

Håkan Sand and Barbara Zimmermann contributed equally to this study and share first authorship. Håkan Sand and Barbara Zimmermann conceived and designed the study. All authors contributed to the design and secured the funding. Barbara Zimmermann analyzed the data. Håkan Sand and Barbara Zimmermann wrote the first draft and all authors contributed to multiple revisions of the manuscript.

## CONFLICT OF INTEREST STATEMENT

The authors declare no conflicts of interest.

## Supporting information


Appendix S1.

Appendix S2.

Appendix S3.

## Data Availability

Data (Zimmermann, 2025) are available on DataverseNO: https://doi.org/10.18710/UWXPHW.
