# Supplementary material for: Quantifying large carnivore predation relative to human harvest on moose in an intensively managed boreal ecosystem
Source: Ecol Appl. 2025 Feb 11;35(1):e70000. doi: 10.1002/eap.70000 (PMC11811747; doi:10.1002/eap.70000)
Supplement: Supplementary file 1 — Appendix S1. [file EAP-35-e70000-s001.pdf]

Ecological Applications

Quantifying large carnivore predation relative to human harvest on moose in an intensively managed boreal ecosystem

Håkan Sand, Barbara Zimmermann, Petter Wabakken, Ane Eriksen, Camilla Wikenros

Appendix S1

Estimating seasonal and annual wolf predation

During the time period 2001 - 2022, we conducted multiple kill rate studies on GPS-collared wolves at different times of the year (Sand et al. 2005, 2008, Wikenros et al. 2023, SKANDULV unpublished data). These studies lasted for 4-16 weeks. We therefore had to extrapolate the kill rate found during the studies to the entire season (summer or winter) by modelling the time interval between consecutive moose kills as a function of the Julian date. We divided the data into summer (June 1 - Oct 9) and Winter (Oct 10 - May 31) and created a Julian date starting at June 1. For both seasons, we transformed the response variable (time interval between kills) by taking the square root to meet the assumption of normality for linear regression. The only fixed variable was Julian date and we retained it in the model if significant ($p < 0.05$). The study-id was entered as a random factor. For the modelling, we used the R-package glmmTMB.

The summer model included data from 13 wolf territories distributed over 25 study periods, most of them conducted during the first half of the summer (Figure S1), The time interval between consecutive moose kills increased throughout the summer (Marginal effect: Time interval = (1.1456 + 0.0058*Julian date)^2, P(slope) < 0.001). We used the model to predict the territory-specific cumulative number of kills throughout the summer season (Table S1). For n = 2 territory-years, we conducted the summer study in the year following the winter study. For territory-years without any summer studies at all (n = 5), we predicted the number of moose kills from the marginal effects of the model (mean = 57 moose kills, 95% CI = 49 – 69).

The winter model included 31 studies, most of them conducted during February – April, but we also did some studies in October-January (Figure S1). The time interval between consecutive moose kills was not related to the Julian date ($p = 0.841$), and we therefore assumed that the time intervals found for a specific study could be extrapolated to the entire study period. This allowed us to estimate the number of moose killed during winter, including the 95% confidence interval estimated from the variation in the time interval between kills for a given study (Table S1).

The annual wolf predation was estimated by summing summer and winter estimates of the territory-years included in this study, and the confidence interval of the annual estimates was calculated from the summed variance of the seasonal estimates (Table S1).

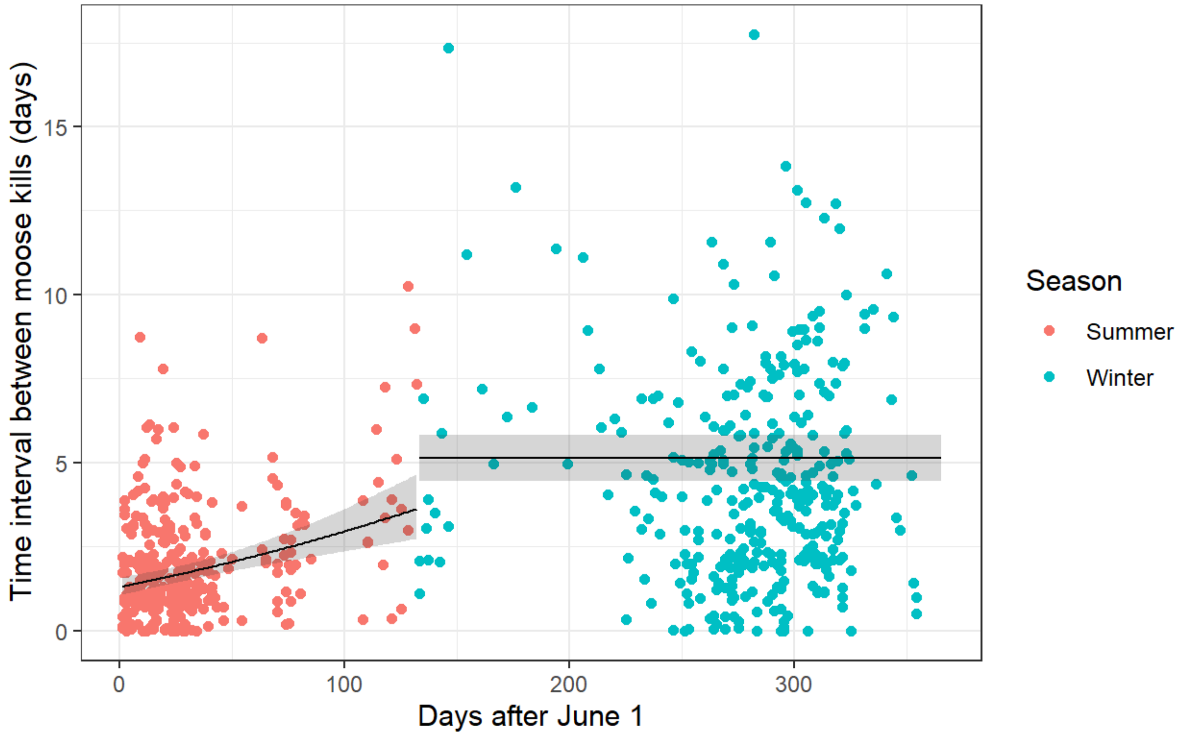

Figure S1: Time interval in days between consecutive moose kills during summer (June 1 – October 9, red) and winter (Oct 10 – May 20, blue) for n = 25 summer and n = 31 winter predation studies, respectively. All data is from field-checks of positions from GPS-collared wolves in Scandinavia, conducted in the time period 2001 – 2022.

Table S1: Wolf territories included in this study, with study year, the length of wolf predation studies in winter and summer, number of squares and sample plots visited to count the number of fecal pellet groups of moose, estimated brown bear density, traffic-related mortality, and estimated number of moose killed by wolves during winter (October 10 – May 31), summer (June 1 – October 9) and for the entire year, with 95% CI. The confidence interval of the yearly number of moose killed by wolves was estimated from the sum of the seasonal variation. Mean and confidence interval of the estimated count of seasonal and yearly kills across studies was estimated from the inverse-variance weighted mean. This table is complementary to the information given in Table 1 in the main text.

Wolf territory	Year of study	Study period winter (N days)	Study period summer (N days)	Number of squares/plots for FPG	Brown bear density (N 1000 km $^{-2}$)	Traffic-related mortality	Wolf-killed moose winter	95% CI	Wolf-killed moose summer	95% CI	Wolf-killed moose year	95% CI
Aspafallet	2015	42	42	41/1491	0.6	13	31	22-50	50	37-72	81	55-118
Bograngen	2003	63	42	82/3047	1.6	17	62	48-89	69	53-95	131	102-162
Djurskog	2004	56	42	42/1559	0.1	2	62	42-115	75	55-106	137	91-181
Fulufjället	2009	52	42	62/2280	19.3	5	35	23-74	74	56-102	109	75-143
Gräsmark	2007	50	-	108/3848	0.5	22	91	70-128	57	49-69	148	117-178
Gråfjell	2002	132	63	116/4159	1.3	20	61	48-81	62	48-82	123	99-146
Jangen	2004	60	-	51/1918	0.6	14	53	40-79	57	49-69	110	88-132
Juvberget	2018	42	57	48/1855	1.8	4	54	36-108	70	51-101	124	81-168
Kloten	2008	50	28	130/1934	0.5	4	61	47-88	56	39-89	117	86-150
Kukumäki	2014	54	77	48/1757	47.9	10	32	19-103	55	40-81	87	20-168
Norrsjön	2018	43	28	66/2317	1.5	10	41	27-90	57	41-85	98	60-137
Norrsjön	2019	44	-	192/6693	1.5	12	61	49-81	55	38-87	116	87-145
Prästskogen	2022	48	28	62/2269	28.9	4	39	31-53	53	39-77	92	70-114
Slettås	2017	73	-	121/604	1.0	4	45	33-70	57	49-69	102	74-124
Tandsjön	2012	84	33	65/2600	31.8	2	41	32-55	50	35-77	91	67-114
Tenskog	2010	57	27	49/1960	41.1	8	36	28-52	63	46-90	99	74-124
Tyngsjö	2002	84	-	96/3840	0.5	5	62	47-91	57	49-69	119	95-143
Ulriksberg	2007	54	-	71/2840	1.1	10	44	36-55	57	49-69	101	87-115
Varåa	2018	42	-	42/1589	1.7	1	23	15-51	53	39-75	76	50-102
Varåa	2019	42	29	38/190	2.2	5	47	36-66	53	39-75	100	76-123
Mean		58.6	41.4	76.8/2437.5	9.3	8.6	46.2		57.7		103.9	
1.96 * SE		9.6	8.7	17.7/632.5	6.7	2.7	3.8		3.4		5.1	
