# Supplementary material for: Quantifying large carnivore predation relative to human harvest on moose in an intensively managed boreal ecosystem
Source: Ecol Appl. 2025 Feb 11;35(1):e70000. doi: 10.1002/eap.70000 (PMC11811747; doi:10.1002/eap.70000)
Supplement: Supplementary file 2 — Appendix S2. [file EAP-35-e70000-s002.pdf]

Ecological Applications

Quantifying large carnivore predation relative to human harvest on moose in an intensively managed boreal ecosystem

Håkan Sand, Barbara Zimmermann, Petter Wabakken, Ane Eriksen, Camilla Wikenros

Appendix S2: Estimation of moose abundance in wolf territories

Estimating moose abundance in wolf territories at June 1 (MStart) from winter fecal pellet group densities and mortalities

Our analyses required an estimate of the number of moose in each wolf territory on June 1, defined here as directly after calving. For this, we relied on moose fecal pellet group (FPG) counts conducted during May in the following spring. The counts included all FPGs accumulated during the winter, from leaf fall in October 10 to the time of counting shortly before the next calving event. However, winter FPG densities do not reflect the number of moose neither at the end nor at the start of the winter sampling interval, because moose that go missing during the winter period will not deposit any FPGs after their death. We therefore needed to calculate how many moose (or FPGs) had gone missing during the winter. These missing FPGs needed to be added to the FPGs we had counted in the field, to get an estimate of the number of FPGs (or moose) at 10 October. Once the moose abundance at 10 October was known, we could add all moose that went missing during summer due to predation from wolves and bears, traffic accidents or other mortality. This results in an estimate of moose abundance at June 1st (MStart).

Because winter mortality is not constant, but may change over time, we used a day-by-day approach that estimated for each day between October 10 in year t to May 31 in year t+1 how many moose (or fractions of moose) were harvested, predated, died in traffic accidents, or died of other causes. We divided this winter period into three time periods: Period 1 October 10-31, period 2 November 1 - December 31, and period 3 January 1 - May 31.

Harvest mortality

The main part of the moose harvest season started on September 25 in Norway and on the second Monday in October in Sweden. Because most studied wolf territories were entirely or partly in Sweden, we assumed that moose harvest started with period 1, i.e. on October 10. We also assumed that 90% of all harvested moose died during the first three weeks of the harvest season, i.e. in time period 1 (Wikenros et al. 2013), because hunters concentrate their activities to the first period, both due to more favorable climate for hunting, and the competition for moose between neighboring hunting teams. We also assumed that harvest decreased linearly during this period of 22 days. The remaining 10% was set to be harvested in period 2 (61 days), again with a linear decrease from November 1 to December 31. We assumed no moose harvest after December 31 (period 3). This is when hunting season stops in Norway. In Swedish territories, harvest season may continue to end of January or even end of February, but the proportion of moose harvested during this coldest period of the season is very low (Wikenros et al. 2013).

The annual harvest per territory was retrieved from harvest statistics, see main text, data in csv-file “Input_1_ moose_start.csv”. We first calculated the number of harvested animals at October 31 and the slope of the linear decrease. This allowed us then to find the intercept and slope of the linear decrease in period 1.

Winter wolf predation mortality

After October 10, there is practically no brown bear predation any longer, so we only considered wolf predation on moose. We assumed a territory-specific, constant kill rate throughout October 10 - May 31, a total of 233 days. See Appendix S1 for the estimation of the total number of moose killed by wolves during winter. The daily kill rate was therefore simply the territory-specific winter kill rate (see main text and Appendix S1 for estimation) divided by 233.

Vehicle-related mortality

See main text for the source of traffic-related mortality in moose, and how we estimated this also for territories where we did not have access to official statistics. Traffic accidents vary during the winter, mostly because the accumulation of snow makes the moose to spend more time in the valley bottoms, where they might cross roads. Our source file of moose-traffic collisions contained the date of collision. This allowed us to calculate how the collisions in all wolf territories were distributed across the different months, i.e. for each month, we had a proportion of the yearly moose collisions (csv-file “Input_2_for_estimation_of_moose_start.csv”). This proportion was used to calculate territory-specific, daily traffic mortalities.

Winter other mortality

For other mortality than human harvest, vehicle collisions, or predation, e.g. diseases, senescence, accidents, we used the same method as Wikenros et al. 2013 (Plos One, page 5). It was based on the assumption that other mortality was about the same size as traffic-related mortality, based on earlier studies (Ericsson & Wallin 2001, Broman et al. 2002). According to Wikenros et al. (2013) we assigned all moose calf and 60 % of adult mortality to the winter months January - April. The other 40% of adult mortality was distributed evenly to May - December (5% per month). We then corrected for compensatory mortality by assuming that 19% of wolf-killed moose calves and 7% of wolf-killed adults were compensatory to other mortality from January to April (Sand et al. 2012, Wikenros et al. 2013). For the other months of the year, we considered that other mortality was mostly additive to harvest, predation and traffic. Age distribution in the moose population during winter was estimated at 30% calves and 70% adults (Sand et al. 2012).

Summing up winter mortality

We then summed all mortality to estimate total mortality for each day and wolf territory between October 10 - May 31.

We then calculated with how many moose-days these moose that died during October 10 - May 31 contributed to the pellet count. The ones dying on 10 October (day 0) did not contribute at all, the ones dying on 11 October contributed with 1 day etc.

In the next operation, we summarized the data across all days of the time period October 10 - May 31, to estimate per wolf territory 1) the total number of moose-days all dead moose had contributed to the pellet count, and 2) Total mortality

We added this information to the input table (Output) and calculated in this way the number of moose still being alive on May 31, and how many were alive before October 10.

Adding summer mortality

We then added the moose that died during summer, between June 1 and Oct 10. We assumed no harvest. For summer predation estimates from wolves and brown bears, see main text and

Appendix S1. For traffic and other mortality, we used the monthly proportions. This allowed us finally to estimate the sum of other mortality after correcting for compensatory mortality during the winter months, see above, and the number of moose present in the wolf territory at the beginning of the moose year at June 1, right after calving (Mstart).

Estimating annual mortality rates

We then estimated the mortality rates (proportion of moose at June 1 that died during the moose year June 1 - May 31), both total and for the different causes of death separately. We also estimated the moose density in the wolf territory at June 1.

References

Broman, E., Wallin, K., Steén, M., & Cederlund, G. (2002). `Mass´deaths of moose Alces alces in southern Sweden: population level characterisation. Wildlife Biology, 8(3), 219- 228.

Ericsson, G., & Wallin, K. (2001). Age-specific moose (Alces alces) mortality in a predator-free environment: Evidence for senescence in females. Ecoscience, 8(2), 157-163.

Sand, H., Vucetich, J. A., Zimmermann, B., Wabakken, P., Wikenros, C., Pedersen, H. C., Peterson, R. O., & Liberg, O. (2012). Assessing the influence of prey–predator ratio, prey age structure and packs size on wolf kill rates. Oikos, 121(9), 1454-1463.

Wikenros, C., Sand, H., Ahlqvist, P., & Liberg, O. (2013). Biomass flow and scavengers use of carcasses after re-colonization of an apex predator. PloS one, 8(10), e77373.
