# Supplementary material for: Quantifying large carnivore predation relative to human harvest on moose in an intensively managed boreal ecosystem
Source: Ecol Appl. 2025 Feb 11;35(1):e70000. doi: 10.1002/eap.70000 (PMC11811747; doi:10.1002/eap.70000)
Supplement: Supplementary file 3 — Appendix S3. [file EAP-35-e70000-s003.pdf]

Ecological Applications

Quantifying large carnivore predation relative to human harvest on moose in an intensively managed boreal ecosystem

Håkan Sand, Barbara Zimmermann, Petter Wabakken, Ane Eriksen, Camilla Wikenros

Appendix S3: AIC table of linear and non-linear univariate regression for predation and harvest rate

Table S1: AICc-table of univariate linear and non-linear regression models used to explain the variation in the estimated wolf predation rate on moose across N = 20 wolf territories in Scandinavia. Explanatory variables were 1) wolf territory size, 2) wolf pack size, 3) wolf density, 4) estimated moose abundance at June 1 (M_{Start}), 5) estimated moose density at June 1, 6) the ratio of moose to wolves, and 7) the estimated annual number of moose killed by wolves. We tested for linear (LIN), logarithmic (LOG), exponential (EXP) and power-function (POW) relationships, calculated McFadden's R^2 and compared these models with the null model (CST) using an ANOVA-test (P).

Predictor	Model	AICc	Delta_AICc	R^2	P
Territory size	LOG	-89.76	0.00	0.68	<0.001
	EXP	-89.64	0.12	0.68	<0.001
	POW	-89.33	0.43	0.68	<0.001
	LIN	-87.04	2.72	0.64	<0.001
	CST	-76.35	13.41		
Pack size	CST	-76.35	0.00		
	POW	-66.90	9.45	0.00	0.832
	LOG	-66.90	9.46	0.00	0.843
	EXP	-66.88	9.47	0.00	0.874
	LIN	-66.88	9.48	0.00	0.883
Wolf density	CST	-76.35	0.00		
	LOG	-74.56	1.79	0.32	0.009
	POW	-74.12	2.23	0.30	0.012
	LIN	-73.15	3.21	0.27	0.019
	EXP	-72.60	3.75	0.25	0.025
Moose abundance	POW	-108.47	0.00	0.88	<0.001
	EXP	-105.88	2.59	0.86	<0.001
	LOG	-105.18	3.29	0.85	<0.001
	LIN	-88.89	19.58	0.67	<0.001
	CST	-76.35	32.12		
Moose density	CST	-76.35	0.00		
	LIN	-70.83	5.52	0.18	0.062
	EXP	-70.82	5.53	0.18	0.062
	LOG	-70.71	5.64	0.18	0.066
	POW	-70.43	5.92	0.16	0.077
Moose-wolf ratio	EXP	-77.88	0.00	0.42	0.002
	LOG	-76.92	0.96	0.40	0.003
	CST	-76.35	1.53		

	POW	-75.75	2.13	0.36	0.005
	LIN	-75.60	2.27	0.35	0.006
Annual kill	CST	-76.35	0.00		
	POW	-68.17	8.18	0.06	0.283
	LOG	-68.14	8.21	0.06	0.287
	LIN	-68.12	8.23	0.06	0.291
	EXP	-68.12	8.23	0.06	0.292

Table S2: AICc-table of univariate linear and non-linear regression models used to explain the variation in the estimated harvest rate on moose across N = 20 wolf territories in Scandinavia. Explanatory variables were 1) wolf predation rate on moose, and 2) total predation rate on moose (wolf and bear). We tested for linear (LIN), logarithmic (LOG), exponential (EXP) and power-function (POW) relationships, calculated R^2 (see main text) and compared these models with the null model (CST) using an ANOVA-test (P).

**Predictor**	**Model**	**AICc**	**Delta_AICc**	**R^2**	**P**
Wolf predation rate	CST	-55.99	0.00		
	EXP	-49.19	6.81	0.13	0.125
	LIN	-49.08	6.92	0.12	0.133
	POW	-48.61	7.38	0.10	0.173
	LOG	-48.43	7.57	0.09	0.193
Wolf and bear predation rate	CST	-55.99	0.00		
	POW	-46.53	9.46	0.00	0.850
	LOG	-46.53	9.46	0.00	0.851
	EXP	-46.52	9.47	0.00	0.875
	LIN	-46.52	9.47	0.00	0.876
